# Simplifying Transcatheter Tricuspid Valve Replacement With 3-Dimensional Intracardiac Echocardiography

**DOI:** 10.1016/j.jscai.2024.101347

**Published:** 2024-03-06

**Authors:** Carlos E. Sanchez, Steven J. Yakubov

**Affiliations:** Department of Interventional Cardiology, OhioHealth Riverside Methodist Hospital, Columbus, Ohio

**Keywords:** transcatheter tricuspid valve replacement, 3D intracardiac echocardiography, tricuspid valve regurgitation, imaging

## Abstract

Transcatheter tricuspid valve replacement (TTVR) offers the potential for improved outcomes for the significant number of patients with severe tricuspid valve regurgitation relative to current treatment options. Imaging is a critical component of the success of this procedure. Here we describe strategies and techniques for the use of 3-dimensional intracardiac echocardiography as an adjunct to standard transesophageal echocardiography during TTVR procedure.

Moderate-to-severe tricuspid valve regurgitation (TR) affects more than 1.6 million people in the United States; among people with isolated primary TR, 5-year mortality approaches 48%.^1^^,^^2^ Despite its frequency and the poor prognosis of these patients, TR has historically been undertreated relative to other valve diseases, in part due to relatively poor outcomes with conventional surgical techniques in this often frail patient population.^3^ Ongoing clinical trials of transcatheter tricuspid valve replacement (TTVR) and tricuspid repair devices suggest that these technologies may improve outcomes relative to typical surgical therapies,^4^, ^5^, ^6^ and the first TTVR system may be approved in the United States as early as mid-2024, marking a likely inflection point in the number of TTVR procedures performed.

Procedural imaging is a critical element of successful transcatheter structural heart procedures. New device technologies for structural heart interventions have become more sophisticated as indications expand, necessitating advanced real-time imaging to ensure optimal outcomes for these more complex procedures. Intracardiac devices, such as TTVR, have primarily relied on transesophageal echocardiography (TEE) imaging with fluoroscopy to analyze anatomic cardiac structures and provide guidance on proper device deployment. However, in the context of tricuspid valve (TV) procedures, it is associated with several challenges. Visualization of the TV by TEE is inherently difficult owing to the variable position of the esophagus in relation to the plane of the TV annulus, which limits the structural definition of the TV leaflet body. Additionally, its structural complexity, the interpatient structural heterogeneity, the relative thinness of its leaflets, and its anterior anatomic location may lead to problems with acoustic shadowing and far-field reverberation, limiting confirmation of TTVR positioning, insertion, and procedural success.^7^

The location, complexity, and dynamic nature of the TV and the limitations of TEE in this setting have led to the increased use of adjunct intracardiac echocardiography (ICE) to better guide procedures. Two-dimensional (2D) ICE catheters are broadly available but are limited by their ability to show only cross-sectional slices of anatomy. Three-dimensional (3D) ICE is an emerging technology that addresses the limitations of 2D ICE and 3D TEE by providing volume data and near-field images that are less impacted by acoustic shadowing or influenced by anatomic considerations, enabling visualization and quantification of target structures and allowing for optimal device sizing and placement during transcatheter TV interventions.^8^

## Strategies and techniques for the adjunctive use of 3D ice for TTVR

3D ICE guidance for TTVR can often improve TV imaging with minimal manipulation of the ICE catheter. It is often beneficial to have an operator who is skillful in reconstructing images, distinct from the operator of the catheter, to facilitate the procedure. This communication is similar to that of the procedural operator with an imaging specialist performing TEE.

**Figure 1:**
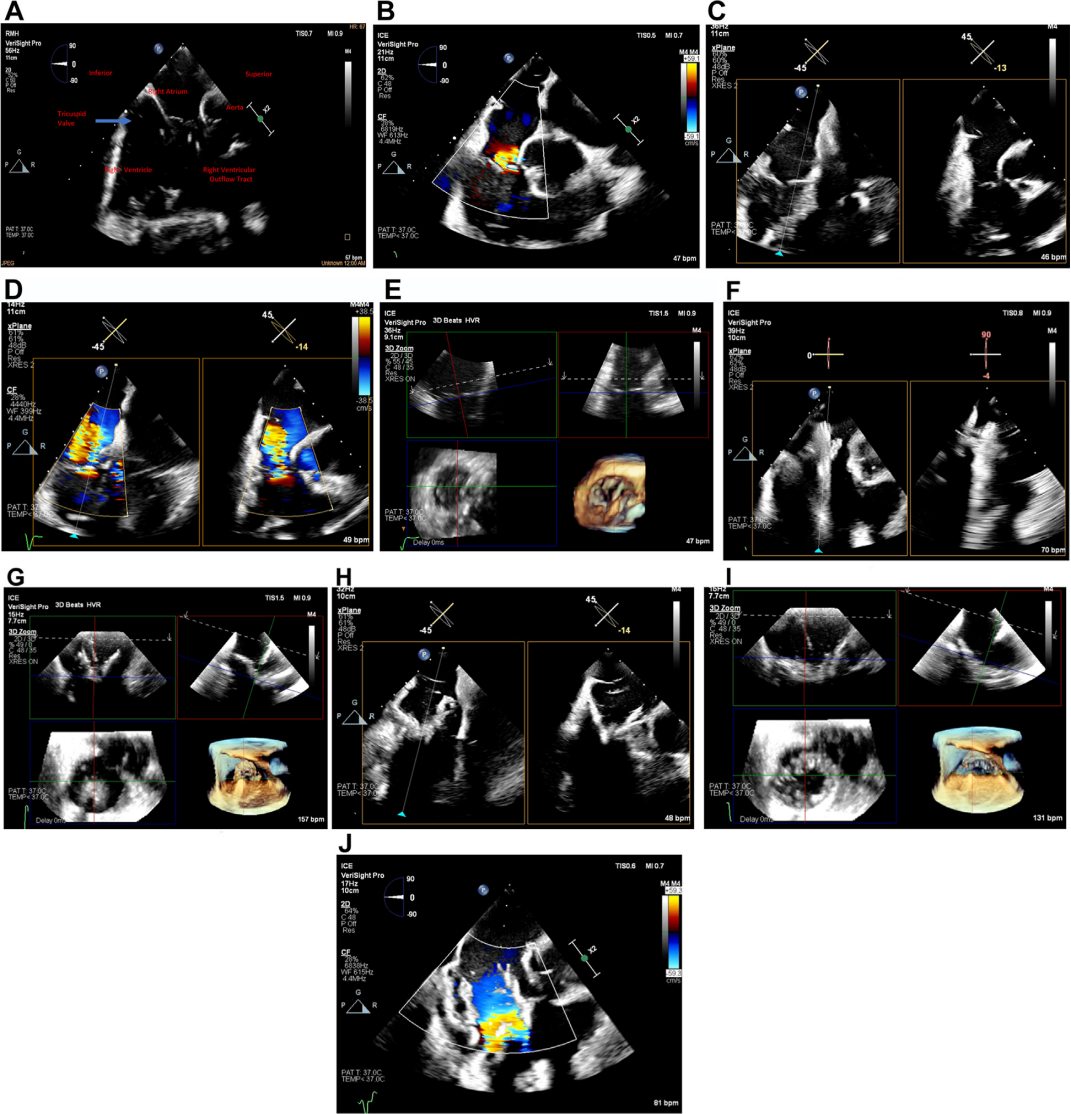
**Intracardiac echocardiography imaging for transcatheter tricuspid valve replacement.** (**A**) 2-dimensional ICE view showing all 3 tricuspid leaflets from home view without and (**B**) with color. (**C**) Right ventricular inflow/outflow biplane view without and (**D**) with color. (**E**) MPR view is obtained by placing the crosshair markers across the TV annulus, creating a 3D en face view of the TV in real time. (**F**) The TV is crossed with the capsule centered on biplane view. (**G**) The TTVR brim is opened above the TV, aligned above the TV parallel with the tricuspid plane, and centered within the annulus on MPR imaging. (**H**) The TTVR is advanced once the position is properly aligned with the annulus. Biplane imaging confirms the brim of the bioprosthesis is coaxially aligned to the TV annulus and (**I**) MPR imaging is used to confirm proper alignment of the bioprosthesis in multiple orthogonal views. (**J**) The TTVR is deployed on a biplane view once positioning is confirmed. Images were obtained using the Philips VeriSight PRO 3D ICE catheter. 3D, 3-dimensional; ICE, intracardiac echocardiography; MPR, multiplane reconstruction; TTVR, transcatheter tricuspid valve replacement; TV, tricuspid valve.

The initial 3D ICE imaging position for the TV is the home view, obtained from the right atrium (RA), with and without color, to identify all 3 TV leaflets and the origination of the TR jet (Figure 1A, B). Biplane images are obtained to determine the area of maximum regurgitation and full anatomic assessment, including a right ventricular inflow/outflow view (Figure 1C, D). 3D multiplanar reconstructions (MPR) are obtained by placing the the region of interest over the TV annulus, creating a 3D en face view of the TV in real time (Figure 1E). Ideally, the aortic valve is placed in the 5 o’clock position, similar to TEE, to help identify all leaflets in a consistent manner according to this anterior landmark. The transfemoral sheath and delivery catheter are positioned in the RA, guided by fluoroscopy and TEE or 3D ICE. The TTVR is steered from the septal side of the RA toward the TV and positioned above the TV annulus.

Using 3D ICE guidance, the TV is crossed with the capsule centered and the height adjusted as required. The anatomic landmarks of the TV annulus are identified.

Orthogonal views with biplane or real-time 3D MPR can be displayed simultaneously (Figure 1F). The TTVR brim is opened above the TV, aligned above the TV parallel with the tricuspid plane, and centered within the annulus (Figure 1G). The TTVR is then advanced to the target implantation position under direct visualization of the TV annulus with the orthogonal views as guidance (Figures 1H, I). If the position of the TTVR is acceptable, the bioprosthesis is advanced, fully deployed, and confirmed fluoroscopically and by 3D ICE once it is fully released (Figure 1J). Using fluoroscopic and echographic guidance, the delivery catheter is withdrawn centrally through the TTVR and into the RA.

The absence of acoustic interference of the TTVR delivery system at the level of the TV annulus is a distinct imaging advantage with 3D ICE. This facilitates proper target implant position, allowing the operator to maintain the delivery system in plane throughout deployment. Crosshair alignment of the image is used to properly orient the TV annulus with the TTVR brim, confirming coaxial insertion and proper engagement with the TV annulus in all orthogonal views. Doppler flow is used to determine residual TV regurgitation.

## Conclusions

Real-time 3D ICE is a novel technology that is useful as an adjunct to TEE to visualize and guide bioprosthesis insertion, position, and deployment in TTVR. Owing to the ability to deflect in multiple planes, simultaneously adjust biplane images along with electronic rotation capabilities, and digitally steer without moving the catheter, 3D ICE has the potential to overcome many of the current imaging limitations of TEE. Current generations of 3D ICE catheters have some limitations, including reduced resolution at certain angles in biplane and MPR modes. Catheter design enhancements, protocols for image guidance, and enhanced experience and education will likely make adjunctive 3D ICE a routine component of TTVR.
